# High-Throughput RNA-Seq Data Analysis of the Single Nucleotide Polymorphisms (SNPs) and Zygomorphic Flower Development in Pea (*Pisum sativum* L.)

**DOI:** 10.3390/ijms18122710

**Published:** 2017-12-20

**Authors:** Keyuan Jiao, Xin Li, Wuxiu Guo, Shihao Su, Da Luo

**Affiliations:** 1 Guangdong Key Laboratory of Plant Resources, School of Life Sciences, Sun Yat-sen University, Guangzhou 510275, China; jiaokeyuan198653@126.com (K.J.); guowx3@mail2.sysu.edu.cn (W.G.); sushihao@mail2.sysu.edu.cn (S.S.); dluo@sibs.ac.cn (D.L.); 2 College of Life Sciences, Laboratory Center of Life Sciences, Nanjing Agricultural University, Nanjing 210014, China

**Keywords:** pea (*Pisum sativum* L.), next-generation sequencing, de novo assembly, zygomorphic flower development, dorsoventral asymmetry, petal-specific gene expression

## Abstract

Pea (*Pisum sativum* L.) is a model plant that has been used in classical genetics and organ development studies. However, its large and complex genome has hindered research investigations in pea. Here, we generated transcriptomes from different tissues or organs of three pea accessions using next-generation sequencing to assess single nucleotide polymorphisms (SNPs), and further investigated petal differentially expressed genes to elucidate the mechanisms regulating floral zygomorphy. Eighteen samples were sequenced, which yielded a total of 617 million clean reads, and de novo assembly resulted in 87,137 unigenes. A total of 9044 high-quality SNPs were obtained among the three accessions, and a consensus map was constructed. We further discovered several dorsoventral asymmetrically expressed genes that were confirmed by qRT-PCR among different petals, including previously reported three *CYC*-like proliferating cell factor (TCP) genes. One MADS-box gene was highly expressed in dorsal petals, and several MYB factors were predominantly expressed among dorsal, lateral, and/or ventral petals, together with a ventrally expressed TCP gene. In sum, our comprehensive database complements the existing resources for comparative genetic mapping and facilitates future investigations in legume zygomorphic flower development.

## 1. Introduction

Pea (*Pisum sativum* L.) has long been regarded as a classical model legume in genetics studies since Mendel’s time. Large collections of mutants in the germplasm make pea a good system for investigating the key components and underlying molecular mechanisms in plant development. For example, large-scale fast neutron (FN) mutagenesis populations were generated from accessions JI2822 and Terese to elucidate the function of genes involved in various biological traits and processes [1,2,3]. Virus-induced gene silencing (VIGS) involving the bean pod mottle virus (BPMV) and pea early browning virus (PEBV) induces gene silencing in accession JI992, thereby allowing the functional analysis of pea and other legumes [1,4,5]. However, based on its large genome (about 4.5 Gb) and high repetitive sequence content, map-based gene cloning is generally difficult to perform in pea.

Genome-wide scanning has been performed in various legume species, including two model legume plants (*Lotus japonicus* and *Medicago truncatula*) and other crop legumes [6,7,8,9]. Papilionoid legumes exhibit extensive genome conservation and share numerous syntenic blocks [10,11,12,13]. Translation genomics is a useful tool for the identification of the important loci in legumes that do not have any available whole genome information [8,14]. In recent years, our studies have also demonstrated that conducting virtual map-based cloning in pea could be an efficient approach in comparative genomic mapping, which is largely dependent on identifying markers in the corresponding regions [2,15]. Next-generation sequencing technology (NGS) has been effectively utilized in large-scale, high-throughput sequencing and construction of comprehensive linkage maps with numerous markers, thereby providing new insights into pea transcriptome [16,17,18,19,20,21,22,23].

One of the key issues in flower development is how floral asymmetry is established. Pea possesses the typical zygomorphic flower that is observed in other species such as the Papilionoid legumes [24]. Dorsoventral (DV) differentiations among three kinds of petals can be distinguished based on their size, shape, and epidermal cell types [1]. However, differentiation can be also manifested in variations in internal (IN) asymmetry among these petals, i.e., the dorsal petals possess IN symmetry, whereas the lateral and ventral petals in pea are asymmetric. Zygomorphic flowers manifest DV asymmetry along the floral plane and IN asymmetry in the organ, and various forms of floral zygomorphy can be found among different plant lineages [1]. In *Antirrhinum majus*, flowers represent a different type of zygomorphy consisting of two asymmetric dorsal petals, two asymmetric lateral petals, together with one symmetrical ventral petal.

The molecular basis of zygomorphy was first studied in detail in *A. majus* [25,26]. Two TCP transcription factors, CYCLOIDEA (CYC) and DICHOTOMA (DICH), which belong to the TEOSINTE BRANCHED1, CYCLOIDEA, and proliferating cell factor (TCP) family, are involved in the control of floral DV asymmetry in *A. majus* [25,26]. Previous studies have demonstrated that eudicot plants recruit TCP genes to establish DV asymmetry through parallel evolution [27,28,29,30]. In legumes, *CYC*-like TCP genes have undergone functional differentiations through multiple duplication events [1,31,32]. In pea, *PsCYC2* and *PsCYC3* confer dorsal and lateral identities, respectively [1]. Unlike *A. majus*, whose IN asymmetry is mainly controlled by DICH [26], our previous study favored an idea that a new locus in pea, *SYMMETRICAL PETALS1* (*SYP1*), is responsible for the establishment of IN asymmetry in both lateral wings and ventral keels [1]. These data suggest that the molecular mechanisms regulating legume floral zygomorphy might be different from *A. majus*, and a transcriptome-wide understanding of genes involved in the morphogenesis of different petals could be an alternative way to address this issue.

To investigate the polymophysim of three accessions, namely, JI2822, Terese, and JI992 and the molecular mechanisms regulating legume floral zygomorphy, high-throughput RNA-Seq data were obtained from 18 samples consisting of different tissues from three accessions and three types of petals. A large number of high-quality SNPs were obtained, and a consensus genetic map was established. Comparative analyses of genetic structures and sequences of pea against *M. truncatula* were also performed. We further found asymmetrically expressed genes in different petals, including one *CIN*-like TCP gene, one MADS-box gene, and several MYB-like factors. Our comprehensive database complements the existing resources in comparative genetic mapping and better facilitates research studies on legume zygomorphic flower development.

## 2. Results

### 2.1. De Novo Assembly of Illumina Paired-End Reads and Unigene Annotation

**Figure 1:**
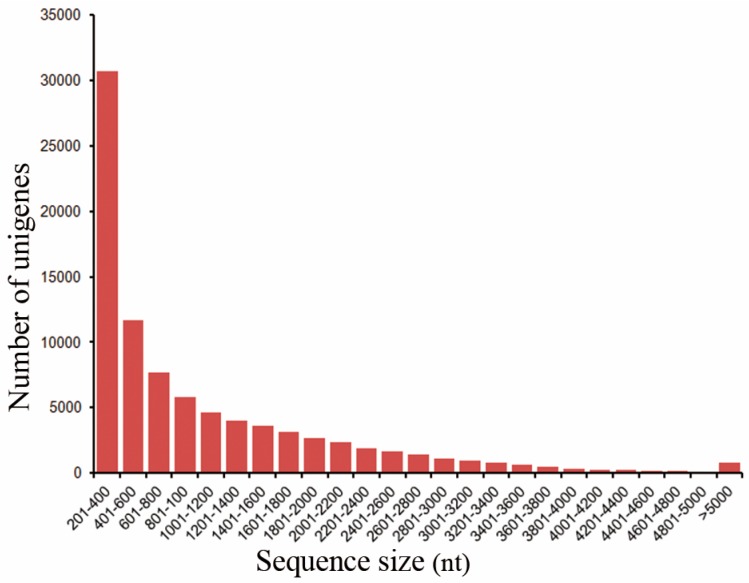
Length distribution of unigenes identified in our de novo assembly Total number (87,137), Total length (91,915,266 bp), Mean length (1054.84 bp), Maximum length (15,111 bp), Minimum length (201 bp), N50 length (1796 bp), N90 length (425 bp), Number of unigenes with >N50 (16,181).

To generate a comprehensive transcriptomic dataset of three accessions, a total of 18 cDNA libraries (Table 1) were produced and were sequenced using the HiSeq 2000 platforms. After stringent quality filtering, a total of 192,977,028; 230,359,898; and 194,019,988 paired-end clean reads with an average read length of 100 bp were obtained from JI2822, Terese, and JI992, respectively. Details of the sequencing outcomes for each tissue-specific library of three accessions are provided in Table 1. The sequence data was deposited to NCBI Sequence Read Archive (SRA, http://www.ncbi.nlm.nih.gov/Traces/sra), with accession number SRP078451. An average of 36.3 million reads was generated per tissue type. The combined sequence length of the Illumina reads was 91.9 Mb and could be de novo assembled into 87,137 high-quality reference unigenes in pea (Figure 1). Sequences of the assembled unigenes ranged from 201 bp to 15,111 bp, with mean sizes of 1054 bp (N50 = 1796, N90 = 425 bp) (Figure 1). A total of 42,337 unigenes (48.59%) were short, with lengths no longer than 600 bp, and 12,363 unigenes (15.22%) were longer than 2000 bp. The unigene length distribution of the assembly is shown in Figure 1. After de novo assembly, all unigenes were then annotated. A total of 47,896 (55%) of the unigenes matched unique protein accessions in the NR database (Table S1).

### 2.2. SNP Identification

All of the sequenced reads from the 18 samples were aligned to the de novo assembled reference unigenes using the BWA v0.7.5a (http://bio-bwa.sourceforge.net/index.shtml) software (set to allow two base mismatches), respectively, and about 66.7% of the total reads can be uniquely matched to reference locations (Table 1). These high-quality reads were then used in detecting SNPs using SAMTools v0.1.19 by individually comparing the three genotypes with the reference sequence, and a total of 9044 high-quality SNPs were identified in 3898 unigenes. Of these, 5918 (in 2813 unigenes), 5132 (in 2528 unigenes), and 5133 (in 2553 unigenes) SNPs were identified among JI2822 and JI992, JI2822 and Terese, and JI992 and Terese, respectively (Table S2).

### 2.3. Comparative Genome Analysis between Pea and Medicago

To further investigation our data, BLAST analysis was performed against 1920 sequences that were previously validated using a GoldenGate assay [18] to generate a consensus genetic map. A total of 1798 unigenes were matched, representing 1254 loci on the pea genetic map. A consensus map comprising a total of 1372 markers (another 118 markers shown in red are SSR markers that commonly occurred in a previous map) [33] was constructed (Figure S1 and Table S3).

**Figure 2:**
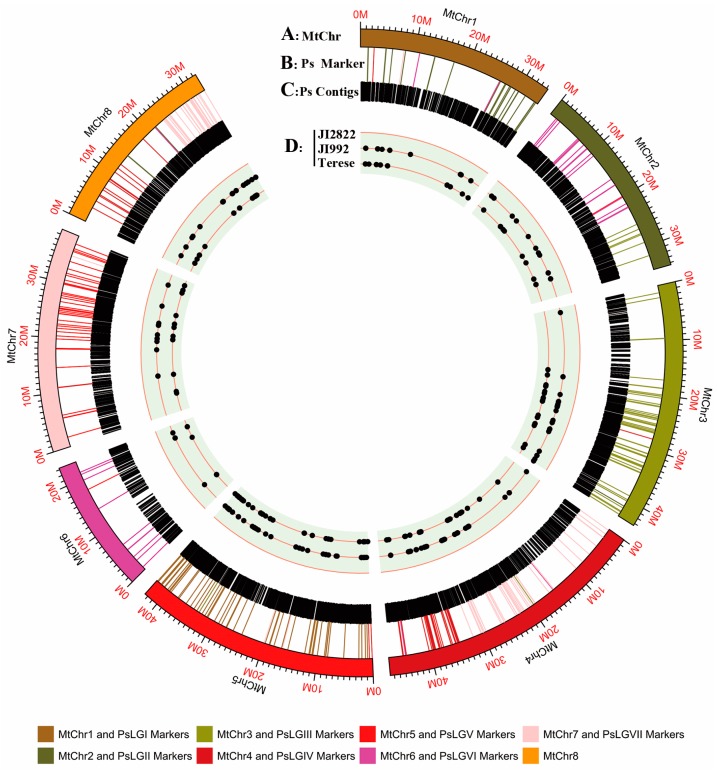
A comparative map between pea and *Medicago.* (**A**) Eight chromosomes of Medicago (Mt). (**B**) 324 unigenes that are located on the consensus map and can be successfully mapped to Mt genome. (**C**) 10,472 unigenes matching to the unique region of the *Medicago* reference genome. (**D**) SNPs among the JI2822, JI992, and Terese pea accessions. Different colors represent various Mt Chrs and PsLGs in (**A**,**B**). Dots in (**D**) represent SNPs among pea accessions JI2822, JI992, and Terese from 324 unigenes in (**B**).

Compared to the *Medicago* (Mt) genome, 10,472 unigenes (12.02% of the total number of unigenes) showed high sequence homology to the unique of the *Medicago* reference genome [34] and were assembled as a virtual chromosome of the pea (Figure 2 and Table S4). Based on the 324 unigenes that were located on the consensus map, comparative mapping indicated macrosynteny between pea and *Medicago*. Among the *M. truncatula* chromosomes, the pea linkage groups PsLGI, PsLGII, PsLGIII, and PsLGV exhibited synteny and colinearity with MtChr.5, MtChr.1, MtChr.3, and MtChr.7, respectively. Conversely, PsLGVI, LGIV, and PsLGVII contained the lowest number of Mt orthologues, indicating complex relationships with the Mt genome (Table S5).

### 2.4. Identification of DEGs in the Three Types of Pea Petals

**Figure 3:**
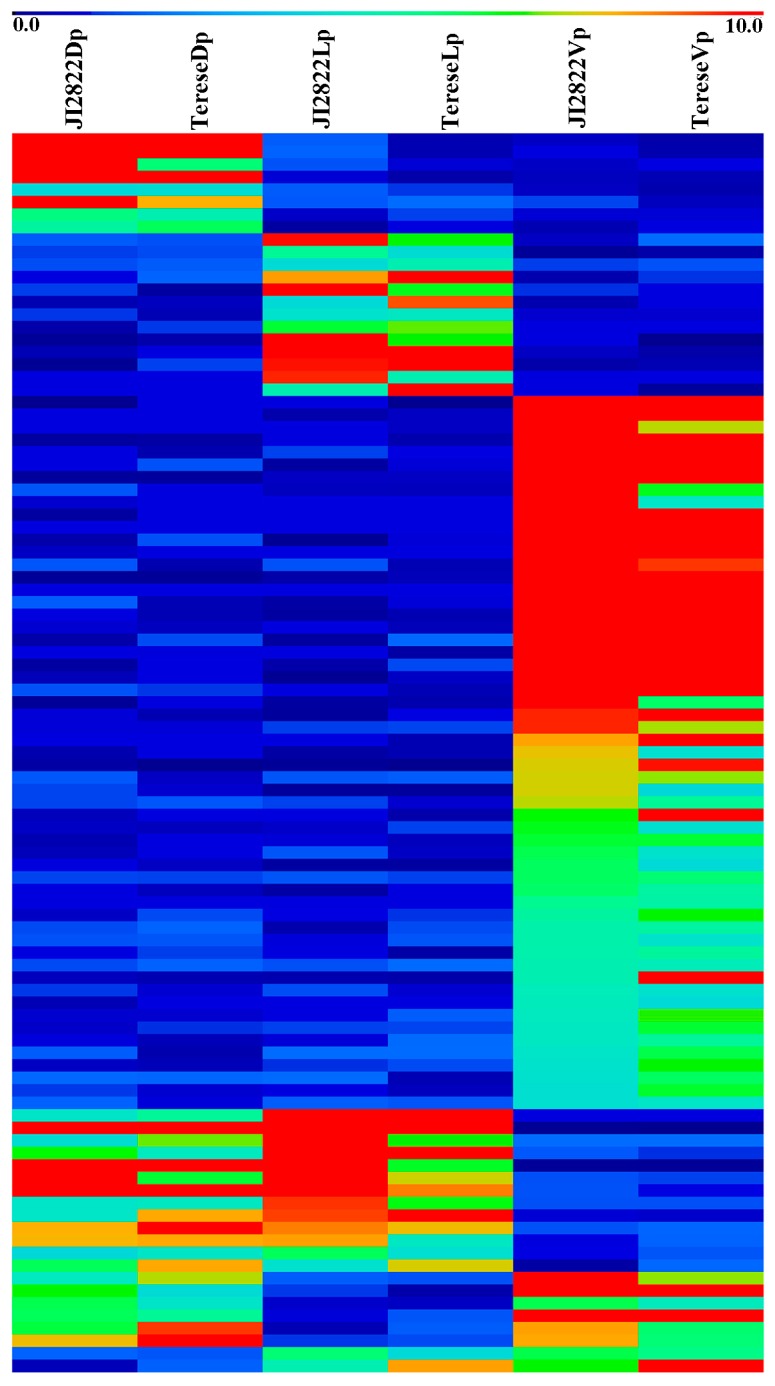
A heat map showing the expression patterns of DEGs in three types of petals. Dp, dorsal petals; Lp, lateral petals; Vp, ventral petals.

To analyze gene expression levels in different tissues or organs, clean reads from 18 samples were individually mapped to the de novo assembly. Only reads that could be uniquely mapped to a one locus of the reference transcriptome were used. Gene expression levels of each sample were expressed as RPKM (Reads Per Kilobase per Million mapped reads) values (Table S1). Expression levels in the dorsal, lateral, and ventral petals were compared against each other. Applying cutoffs of a |log_2_ratio| ≥ 1 and FDR ≤ 0.001, 2080, 2737, and 6345 unigenes were identified, thus primarily comprising 7820 DEGs (9.41% of the total number of unigenes) among the three types of petals (Table S6). Then, combined with the other criteria, namely, one petal’s RPKM ≥ 4.0, and another two petals’ RPKM ≤ 2.0, or two petals’ RPKM ≥ 4.0, and another one’s RPKM ≤ 2.0 and, in order to avoid the influence of different ecotypes, only those genes which met the filter conditions in both JI2822 and Terese backgrounds were selected for further analysis. A set of 8, 13, and 57 DEGs were thus identified as preferentially expressed in the dorsal, lateral, and ventral petals, respectively, and a set of 13, 6, and 2 unigenes showed specific expression patterns in the dorsal-lateral, dorsal-ventral, and lateral-ventral, petals, respectively (Figure 3 and Table S7).

### 2.5. Determination of Gene Categories That Were Differentially Expressed in the Petals

To explore the transcriptional regulation of genes expressed in the three types of petals, we analyzed the RNA-Seq data using BLAST searches (E-value, 1 × 10^−5^) against the public database using DEGs. The DEGs identified under more stringent conditions, including TCP, MADS, and MYB family of transcription factors, may contribute to morphological differences in petals (Table S7).

**Figure 4:**
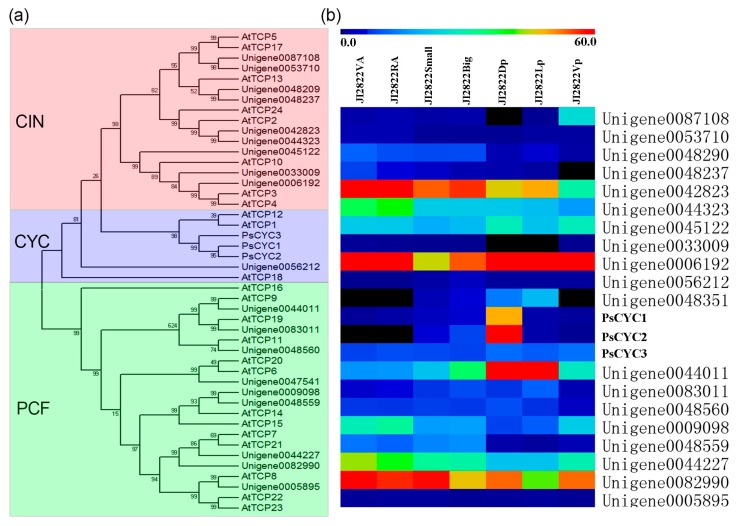
A Neighbor-joining consensus tree and the expression patterns of TCP genes in pea. (**a**) An unrooted protein tree for the TCP proteins using MEGA 5.0.1. The tree summarizes the evolutionary relationships among the 24 AtTCPs and 22 PsTCPs. Bootstrap values are denoted above the nodes. (**b**) The expression patterns of the TCP genes among different organs in pea. VA, Vegetative shoot apices; RA, Reproductive shoot apices; Small, 2-mm small buds; Big, 5-mm big buds; Dp, Dorsal petals; Lp, Lateral petals; Vp, Ventral petals.

TCP transcription factors are widely involved in vegetative and reproductive organ development and are essential in the establishment of flower zygomorphy [35]. To investigate the relationship of the TCP genes between pea and *Arabidopsis*, 22 TCP genes were identified in pea and then compared to 24 TCP genes in *Arabidopsis*. All the TCP factors were grouped into three clades, namely, CIN, CYC/TB1, and PCF (Figure 4a). There were nine members in the CIN clade, four in the CYC/TB1 clade, and nine in the PCF clade. Furthermore, the expressional patterns of these TCP genes in pea were analyzed. 

Consistent with previous studies, *PsCYC1* (Unigene0018426) and *PsCYC2* (Unigene0018425) were preferentially expressed in the dorsal petals, and *PsCYC3* (Unigene0048351) was preferentially expressed in dorsal and lateral petals (Figure 4b). Interestingly, we found a *CIN*-like TCP gene (Unigene0087108) that was preferentially expressed in the ventral petals, suggesting its possible role in the development of pea floral zygomorphy, similar to its homologs identified in *Petrocosmea glabristoma* and *P. sinensis* [36]. The other members in the CIN and PCF clades exhibited no distinct expression patterns among these tissues/organs (Figure 4b).

**Figure 5:**
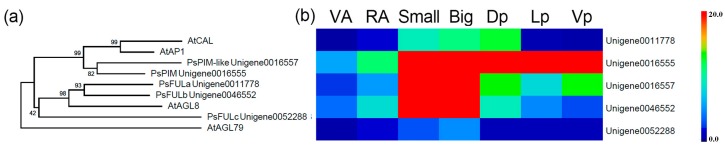
A neighbor-joining consensus tree and the expression patterns of the pea AP1/FUL clade MADS-box genes. (**a**) An unrooted protein tree for the AP1/FUL clade MADS-box genes using MEGA 5.0.1. Bootstrap values are denoted above the nodes. (**b**) The expression patterns of the AP1/FUL clade genes in different organs in pea. VA, Vegetative shoot apices; RA, Reproductive shoot apices; Small, 2-mm small buds; Big, 5-mm big buds; Dp, Dorsal petals; Lp, Lateral petals; Vp, Ventral petals.

One closely related MADS-box gene, *PsFULa* (Unigene0011778), which belongs to the AP1/FUL clade [37], shows distinct expression patterns in the pea petals (Figure 5a). *PsFULa* is predominantly expressed in the dorsal petal, whereas other MADS-box genes in the same clade are evenly or expressed at low levels in all the petals (Figure 5b and Table S7)

Several MYB family genes were also differentially expressed along the DV axis in petals. A *MIXTA*-like gene (Unigene0019000) was upregulated in the dorsal and ventral petals compared to the lateral petals (Table S7). Another MYB gene (Unigene0031178), the homolog of the *LATERAL ORGAN FUSION1* (*LOF1*) and *LOF2* in *Arabidopsis* [38], was upregulated in dorsal petals other than in the lateral and ventral petals (Table S7). One MYB transcription factor, RADIALIS (RAD), acts as the putative downstream target of CYC and DICH by interacting with another MYB-like protein, DIVARICATA (DIV), thereby regulating floral zygomorphy in *A. majus* [39,40]. However, the expressional levels of neither *RAD*-like nor *DIV*-like genes in pea showed significant changes among these three petals (Table S7).

### 2.6. Expression Analysis of Key DEGs in Petals via qRT-PCR

**Figure 6:**
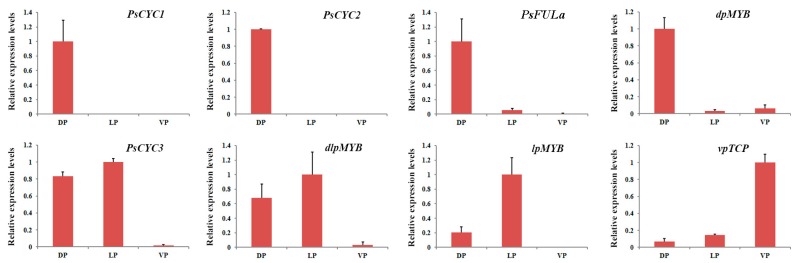
qPCR validation of selected genes with a DV asymmetrical expression patterns in three types of pea petals. *PsCYC1* (Unigene0018426), *PsCYC2* (Unigene0018425), *PsFULa* (Unigene0011778), *dpMYB* (Unigene0031178), *PsCYC3* (Unigene0048351), *dlpMYB* (Unigene0005297), *lpMYB* (Unigene0082746), and *vpTCP* (Unigene0087108). Error bars indicate ±SD.

To validate the expression of these aforementioned genes, qRT-PCR analysis of the three types of petals from young floral buds of JI2822 was performed. We selected eight DEGs (Table S8), which showed expression patterns that coincided with the observed RPKM values from our transcriptome analysis (Figure 6), confirming our transcriptomic data validity.

## 3. Discussion

### 3.1. SNP Variations in Pea and Comparative Genome Analysis

Next-generation sequencing allowed rapid SNP discovery and genotyping in pea [17,18,19,41]. SNP frequencies in plant genomes significantly vary among populations and the status of the analyzed regions [17]. In the present study, based on a transcriptomic dataset, 5918, 5132, and 5133 SNPs were identified among JI2822 and JI992, JI2822 and Terese, and JI992 and Terese, respectively (Table S2). Furthermore, a total of 9044 high-quality SNPs were identified in 3898 unigenes among these accessions, and a consensus map was constructed (Table S2). 

Previous studies have shown that the structure of eight chromosomes of *M. truncatula* and seven linkage groups of field pea are highly conserved (Figure 2) [10,11,42]. The present study have generated substantially more information on different legume genomes, which may be beneficial to future comparative map-based genetic mapping and gene cloning of genes such as *SYP1* in pea [1,2].

### 3.2. Floral Zygomorphy as Revealed by Transcriptional Profiling in Pea

Our previous work showed that zygomorphic flower development depends on the activation of three *CYC*-like TCP factors in pea [1]. To globally understand the regulatory mechanism underlying legume floral zygomorphy, we screened our transcriptome database, which identified eight unigenes that were uniquely expressed in the dorsal petals, similar to *PsCYC1* and *PsCYC2* (Table S7). On the other hand, 13 DEGs were specifically expressed in both dorsal and lateral petals, similar to the expressional pattern of *PsCYC3* (Table S7), indicating potential downstream targets of these *PsCYCs*.

Besides the three early reported CYC/TB1 clade *PsCYCs* genes, we found a *CIN*-like TCP gene (Unigene0087108) that is preferentially expressed in the ventral petals (Figure 4a,b). In *P. sinensis*, Two *CIN*-like TCP genes play key roles together with *CYC*-like TCP genes in the control of floral zygomorphy [36]. In addition, a non-conventional *TB1*-like TCP gene that lacked the R domain controls the floral zygomorphy in rice [43]. These findings suggest that other TCP genes other than *CYC*-like might be involved in floral zygomorphy in pea.

MADS-box proteins are transcription factors that control a wide range of developmental processes in plants [44]. In orchid, MADS-box genes are differentially expressed in various floral organs during perianth formation, resulting in a complex containing different AP3/AGL6 homologs that competitively determine perianth patterns within its flowers [45,46,47]. In the snapdragon, the maintenance of *CYC* in the second whorl requires the existence of MADS-box protein [48]. Similarly, CYC could also be maintained with the expression of PLENA, a C-class MADS factor [48]. One MADS-box gene, *PsFULa*, exhibited a distinct expression pattern in the petals (Figure 5b), suggesting a possible function in regulating floral zygormorphy in pea. It will be an interesting issue to study the timing of expression of these factors and its interaction with PsCYCs in pea, which will help us to understand how these factors orchestrate to establish zygormorphic flower development of the legume species.

MIXTA transcription factors are essential to epidermal cell differentiation [49,50,51,52]. Some unique epidermal cells in the petals of legume plants have been regarded as micro-morphological markers of petal identity [1,32,53]. In the present study, several MYB genes with distinct expressional patterns in petals were identified. A *MIXTA*-like gene (Unigene0019000) was highly expressed in dorsal and ventral petals compared to the lateral petals (Table S7). The MYB protein, RAD, which acts as a downstream target of CYC and DICH, antagonizes the activity of another MYB-like protein DIV to control floral zygomorphy in *A. majus* [39,40]. However, neither the expression level of *RAD*-like genes nor that of *DIV*-like genes in pea shows significant differences among the three petals, suggesting that *RAD*-like MYB genes may not respond to *PsCYCs*, which is similar to that observed in *Arabidopsis*, whose endogenous *RAD*-like genes are not activated by *CYC* [54]. In the future, the expression patterns of the identified MYB genes in the mutants of *PsCYC2* or *PsCYC3* and the double mutants should be examined in more details.

IN asymmetry is another important trait associated with zygomorphy, including the internal asymmetry of the lateral and ventral petals in pea. Several loci such as *SYP1* acting in the same genetic pathway have been identified in pea [2]. *SYP1* establishes the internal asymmetry of the petals, and its mutant give rise to symmetric petals [1]. A number of DEGs are specifically expressed in the lateral or ventral petals (Table S7), indicating that these could be candidate of *SYP1* or its targets.

## 4. Materials and Methods

### 4.1. Plant Materials and RNA Extraction

The plants were grown in a greenhouse at 20 ± 2 °C with a 16-h light/8-h dark photoperiod under 200 μmol·m^−2^·s^−1^ light densities. Eighteen samples were collected at different developmental stages from three accessions, including vegetative shoot apices (from one-week-old seedlings), reproductive shoot apices (from three-week-old plants), 2-mm floral buds, 5 mm floral buds from JI2822, JI992, Terese, and three type petals (dorsal petals, lateral petals, and ventral petals) from 5-mm floral buds of the JI2822 and Terese accessions respectively. Plant total RNA was isolated from different organs/tissues using an RNeasy plant mini kit (Qiagen, Hilden, Germany). RNA quantity and quality were examined using a NanoDrop ND 1000 (NanoDropTechnologies, Wilmington, DE, USA).

### 4.2. Illumina Sequencing and De Novo Assembly

For Illumina sequencing, the poly(A) mRNA was fragmented into small pieces. First-strand cDNA synthesis was conducted using random hexamer primers and reverse transcriptase (Invitrogen, Shanghai, China). Second-strand cDNA was synthesized using RNase H (Invitrogen) together with DNA polymerase I (New England BioLabs, Ipswich, MA, USA). We further constructed 18 cDNA libraries and sequenced the cDNA on an Illumina HiSeq 2000 platform according to the provided manual. For de novo assembly, first, the duplicated reads and low-quality reads were removed. This yielded 617,356,914 clean paired-end reads (Table 1). Unigenes were then de novo assembled using Trinity [55] through all of the seven RNA-Seq samples in the wild-type JI2822 background, respectively. We then used CAP3 to further assemble overlapping regions from a pool of all contigs from each of the RNA-Seq assemblies [56]. The resulting cap3 contigs and singlets were combined together and those that were smaller than 200 nucleotides were eliminated. A total of 192,977,028 clean reads were used during the assembly and this yielded 87,137 unigenes. The distribution of the de novo assembled unigenes is presented in Figure 1.

### 4.3. SNP Discovery

The high-quality filtered reads were mapped to the reference transcriptome using BWA v0.7.5a (http://bio-bwa.sourceforge.net). The resulting mapping data were filtered to identify the reads that mapped to only one position in the reference transcriptome. FastQC v0.10.1 (http://www.bioinformatics.babraham.ac.uk) was used for nucleotide base quality filtration. SAMTools v0.1.19 was used to assess coverage. The high-quality filtered reads were used in the identification of SNPs using SAMTools v0.1.19 by individually comparing the three genotypes to the reference sequence. The identified SNPs were filtered based on following criteria: (1) minimum depth = 10; and (2) all mapped reads support a homozygous genotype. The identified SNPs are presented in Table S2.

### 4.4. Comparative Genome Analysis and the Pea Consensus Map

The de novo assembled unigenes were mapped to genome assemblies of *M. truncatula*, v3.5 (http://www.medicago.org) using Blat v3.2.1, and the mapping data was then filtered to characterize the unigenes that were mapped to only one position in the *M. truncatula* genome. Mapped regions showing ≤50% of each unigene were excluded from further analysis. For the *P. sativum* consensus map, all assembled unigenes were subjected to BLAST analysis against 1340 markers that were previously identified in a pea linkage group [18]. The E-value was 1 × 10^−15^, with a minimum sequence identity of 80%.

### 4.5. Functional Annotation

Sequences were annotated based on various protein databases, including Nr, Swiss-Prot, KEGG, and COG, with an E-value less than 1 × 10^−5^. The Blast2GO program [57] was used in GO annotation of each unigene. The RNA-Seq data of the 18 cDNA libraries were further analyzed for quantification independently according to the RNA-Seq protocol. All the raw files were generated on an Illumina HiSeq 2000 platform.

### 4.6. Phylogeny Analysis

The amino acid sequences were aligned using MEGA 5.0.1. The phylogenetic trees were built using Neighbori-joining methods with 1000 bootstraps replicates. The bootstrap values were denoted above the nodes.

### 4.7. Quantitative RT-PCR

Total RNA was extracted from target tissues as previously described. First-strand cDNA synthesis was performed as described in the manual using 1 μg of total RNA (TaKaRa Biotechnology, Dalian, China). Quantitative RT-PCR (qRT-PCR) was performed using the Roche LightCycler 480 (Roche, Switzerland) and Power SYBR Green Master mix (Applied Biosystems, Foster City, CA, USA), according to the protocols. *PsACTIN* was used as internal control, and the relative expression of each gene in different samples was calculated and measured using the 2^ΔΔ*C*t^ method.

### 4.8. Differentially Expressed Gene (DEG) Analysis

The number of reads per kilobase of exon region in a gene per million mapped reads (RPKM) was used to present gene expression levels [58]. DEGs genes were identified among the dorsal, lateral, and ventral petal libraries by conducting a statistical analysis of the frequency of each transcript and their corresponding *p*-values as described by Audic and Claverie [59]. The significance threshold of the *p*-values in multiple tests was set based on a false discovery rate (FDR). We used “FDR ≤ 0.001 and the absolute value of |log_2_ratio| ≥ 1” as the threshold to judge the significance of gene expression differences. The heat map images were generated using the Microarray Experiment Viewer (MeV) v4.9.0 [60].

## 5. Conclusions

To summarize, our comprehensive database complements the existing resources for comparative genetic mapping in legumes and raises a global overview of the regulatory mechanism underlying legume zygomorphic flower development.

## Figures and Tables

**Table 1:** RNAseq libraries analyzed in this study.

Sample	Total Number of Reads	Number of Reads Showing Unique Matches	Percentage
JI2822 Vegetative shoot apices (VA)	32,214,992	22,922,794	71.16%
JI2822 Reproductive shoot apices (RA)	20,576,714	15,127,796	73.52%
JI2822 2-mm floral buds (Small)	29,167,060	20,502,301	70.29%
JI2822 5-mm floral buds (Big)	33,818,056	24,224,028	71.63%
JI2822 Dorsal petals (Dp)	31,476,682	22,061,407	70.09%
JI2822 Lateral petals (Lp)	22,633,942	15,691,372	69.33%
JI2822 Ventral petals (Vp)	23,089,582	16,568,145	71.76%
Terese Vegetative shoot apices (VA)	26,326,994	17,622,043	66.94%
Terese Reproductive shoot apices (RA)	38,704,866	26,011,448	67.20%
Terese 2-mm floral buds (Small)	41,799,160	28,426,856	68.01%
Terese 5-mm floral buds (Big)	52,957,978	36,260,749	68.47%
Terese Dorsal petals (Dp)	21,246,110	14,305,314	67.33%
Terese Lateral petals (Lp)	24,931,766	16,559,528	66.42%
Terese Ventral petals (Vp)	24,393,024	16,574,182	67.95%
JI992 Vegetative shoot apices (VA)	42,342,066	28,572,173	67.48%
JI992 Reproductive shoot apices (RA)	56,082,530	24,545,471	43.77%
JI992 2-mm floral buds (Small)	47,278,346	32,007,646	67.70%
JI992 5-mm floral buds (Big)	48,317,046	33,808,043	69.97%
Total	617,356,914	411,791,296	66.70%

## Supplementary Materials

Supplementary materials can be found at www.mdpi.com/1422-0067/18/12/2710/s1.
