# Supplementary material for: High-Throughput RNA-Seq Data Analysis of the Single Nucleotide Polymorphisms (SNPs) and Zygomorphic Flower Development in Pea (Pisum sativum L.)
Source: Int J Mol Sci. 2017 Dec 20;18(12):2710. doi: 10.3390/ijms18122710 (PMC5751311; doi:10.3390/ijms18122710)
Supplement: Supplementary file 1 [file ijms-18-02710-s001.zip › Supporting information.pdf]

Supporting information

Table S1 The annotation and RPKM of 87,137 reference unigenes in pea

Table S2 The SNPs among JI2822, JI992, and Terese

Table S3 The 1,798 unigenes representing 1,254 loci on the pea consensus map

Table S4 The 10,472 unigenes that were detected with high sequence homology, matching to the unique unigenes of the Medicago genome

Table S5 The 324 unigenes that were located on the consensus map and identified by comparative mapping of pea relative to *Medicago*

Table S6 The primary DEGs that were identified among three types of pea petals

Table S7 The DEGs preferentially expressed in dorsal, lateral, ventral, dorsal-lateral, dorsal-ventral, and lateral-ventral petals of pea

Table S8 The primers used in the qRT-PCR validation of the DEGs identified in this study

Figure S1 Consensus genetic map of pea

The markers shown on the pea genetic map were based on previously reported map positions [18]. Markers shown in red are SSR markers from a published consensus map [33].
