# Supplementary figures and images for: High-Throughput RNA-Seq Data Analysis of the Single Nucleotide Polymorphisms (SNPs) and Zygomorphic Flower Development in Pea (Pisum sativum L.)
Source: Int J Mol Sci. 2017 Dec 20;18(12):2710. doi: 10.3390/ijms18122710 (PMC5751311; doi:10.3390/ijms18122710)

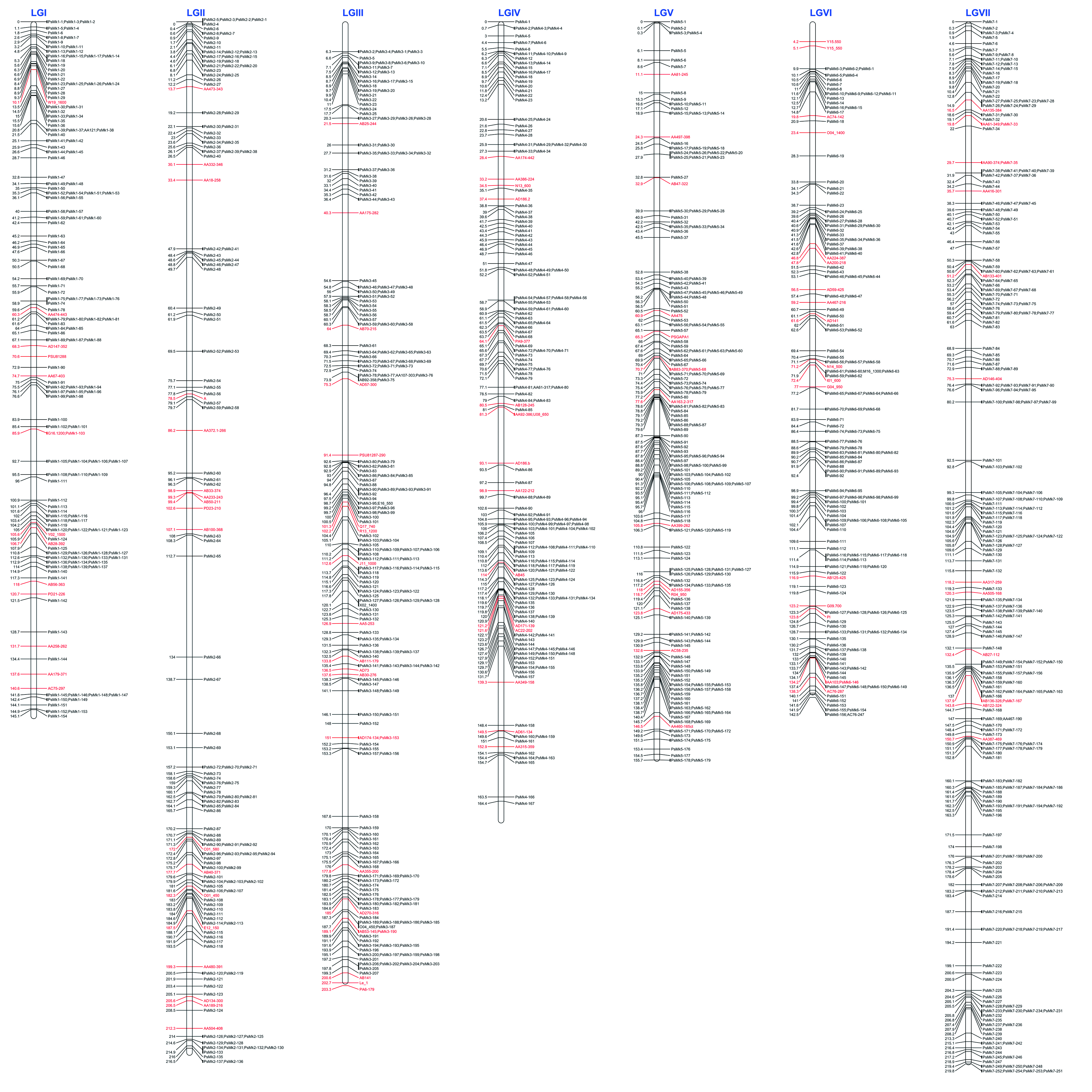

Supplement: Supplementary file 1 [file ijms-18-02710-s001.zip › Figure S1.jpg]
